# Luteolin Causes 5′CpG Demethylation of the Promoters of TSGs and Modulates the Aberrant Histone Modifications, Restoring the Expression of TSGs in Human Cancer Cells

**DOI:** 10.3390/ijms23074067

**Published:** 2022-04-06

**Authors:** Sreepoorna Pramodh, Ritu Raina, Arif Hussain, Sali Abubaker Bagabir, Shafiul Haque, Syed Tasleem Raza, Mohammad Rehan Ajmal, Shalini Behl, Deepika Bhagavatula

**Affiliations:** 1 Department of Life and Environmental Sciences, College of Natural and Health Science, Zayed University, Dubai P.O. Box 19282, United Arab Emirates; sreepoorna.unni@zu.ac.ae; 2 School of Life Sciences, Manipal Academy of Higher Education—Dubai Campus, Dubai P.O. Box 345050, United Arab Emirates; ritu123456@gmail.com (R.R.); behl.shalini@gmail.com (S.B.); b_deepika_18@yahoo.co.in (D.B.); 3 Department of Medical Laboratory Technology, Faculty of Applied Medical Sciences, Jazan University, Jazan 45142, Saudi Arabia; sbagabir@jazanu.edu.sa; 4 Research and Scientific Studies Unit, College of Nursing and Allied Health Sciences, Jazan University, Jazan 45142, Saudi Arabia; shafiul.haque@hotmail.com; 5 Faculty of Medicine, Bursa Uludağ University Görükle Campus, Nilüfer, Bursa 16059, Turkey; 6 Department of Biochemistry, Era’s Lucknow Medical College and Hospital, Lucknow 226001, India; tasleem24@gmail.com; 7 Department of Biochemistry, Faculty of Sciences, University of Tabuk, Tabuk 71491, Saudi Arabia; ajmal.rehan@rediffmail.com

**Keywords:** DNA methylation, luteolin, antiproliferation, antimigration, histone modification

## Abstract

Cancer progression is linked to abnormal epigenetic alterations such as DNA methylation and histone modifications. Since epigenetic alterations, unlike genetic changes, are heritable and reversible, they have been considered as interesting targets for cancer prevention and therapy by dietary compounds such as luteolin. In this study, epigenetic modulatory behaviour of luteolin was analysed on HeLa cells. Various assays including colony forming and migration assays, followed by biochemical assays of epigenetic enzymes including DNA methyltransferase, histone methyl transferase, histone acetyl transferase, and histone deacetylases assays were performed. Furthermore, global DNA methylation and methylation-specific PCR for examining the methylation status of CpG promoters of various tumour suppressor genes (TSGs) and the expression of these TSGs at transcript and protein level were performed. It was observed that luteolin inhibited migration and colony formation in HeLa cells. It also modulated DNA methylation at promoters of TSGs and the enzymatic activity of DNMT, HDAC, HMT, and HAT and reduced the global DNA methylation. Decrease in methylation resulted in the reactivation of silenced tumour suppressor genes including *FHIT, DAPK1, PTEN, CDH1, SOCS1, TIMPS, VHL, TP53, TP73*, etc. Hence, luteolin-targeted epigenetic alterations provide a promising approach for cancer prevention and intervention.

## 1. Introduction

Cancer chemoprevention is the use of compounds that are natural, synthetic, or chemical to reverse, retard, or prevent the onset of cancer using mechanisms that kill malignant cells selectively and spare normal cells [1,2]. These cancer chemopreventive agents can act at all three stages of cancer development—namely, initiation, promotion, and progression. FDA approved chemopreventives such as tamoxifen and raloxifene have the major side effect of causing blood clotting; similarly, tamoxifen—if given for prolonged period—can lead to endometrial cancer. Hence, researchers are shifting their focus towards plant-derived chemopreventive agents, as they have few or no side effects, are more affordable, easily available, and well tolerated. Studies indicate that dietary bioactive compounds such as polyphenols, isothiocyanates, etc. are found to have a profound effect on cancer cells and act via targeting various hallmarks of cancer, leading to the reversal of carcinogenesis [1,3,4]. Various phenolic agents such as EGCG (epigallocatechin gallate), curcumin, etc. have been reported to exert antiproliferation, apoptosis-inducing, and antioxidant properties and to restore the function of TSGs by targeting various molecular targets and pathways [1,4,5,6].

Cumulative data, both experimental and epidemiological, approves the utilization of dietary phytochemicals in chemoprevention, while suggesting that regular consumption of these phytochemicals is a promising new method for cancer prevention [7]. Evidence from many scientific reports have indicated that various bioactive compounds found in vegetables and fruits influence epigenetic modulations by regulating crucial processes that entail tumour suppressor genes reactivation, oncogene suppression, cell-survival protein activation, and apoptosis induction in various cancers [8]. Several nutrients in our daily dietary intake play an important role in DNA methylation either by modulating (DNA methyl transferase) DNMT expression or by altering the presence of methyl donors [8]. Dietary bioactive compounds such as curcumin, tea polyphenols, sulforaphane (SFN), genistein, resveratrol, and others have been found to be effective against cancer cells by altering regulatory mechanisms that directly affect the epigenome [9]. Bioactive agents act upon cancer progression and reverse the process by interfering with the action of (histone deacetylases) HDACs, DNMTs, and HATs (histone acetyl transferase) [8,9,10,11]. Published reports of polyphenol show that they are able to modify chromatin structure and reactivate silenced genes [12,13], potentially reverting malignancy-associated epigenetic alterations in cell lines [2,14]. Hence, modulation of epigenetic mechanisms poses a prominent target for cancer therapeutics [15]. This multidimensional molecular approach of polyphenols can be substantially highlighted for cancer prevention and therapy. Polyphenols have shown the inhibition of proliferation, invasion, and modulation of epigenetic pathways in different cell lines [16,17,18].

Luteolin, a flavone, has been studied as an anticancer agent and is known to inhibit tumour development in cancer cells by inducing apoptosis, inhibiting metastasis by modulation of different pathways such as AKT, MAPK, etc. [19,20,21,22,23,24]. Luteolin has also shown anti-invasive and epigenetic modulatory action such as modulation of methylation and inhibition of HDAC on different cell lines [17,25,26]. In addition to this, flavones have shown a remarkable safety profile (zero toxicity at up to 140 g/day) with no adverse side effects [21]. However, few studies are available on epigenetics modulation such as DNA methylation and histone modifications by DNMT, HMT, and HDAC after luteolin treatment [25,27,28]. Hence, this study was intended to provide experimental evidence of luteolin-mediated changes, targeting its antimigratory and chromatin modulatory nature on HeLa cells for cancer prevention and treatment.

## 2. Results

### 2.1. Luteolin Reduces Methylation of Promoter Tumour Suppressor Genes

**Figure 1:**
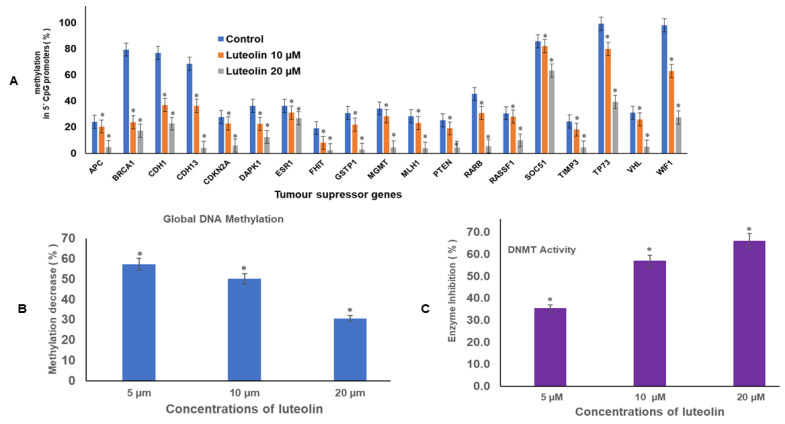
(**A**). Luteolin treatment of HeLa cells at 10 and 20 µM for 48 h demonstrated profound decrease in percentage methylation in 5′ CpG promoter regions of TSGs, as compared with the untreated controls, in a dose-dependent manner (* represents that data is statistically significant with *p* ≤ 0.05). (**B**). Luteolin decreased global DNA methylation in HeLa cells. The data are presented as mean of three independent experiments ± SD (* represents that data is statistically significant with *p* ≤ 0.05). (**C**). Luteolin decreased DNMT activity in HeLa cells in a concentration-dependent manner. The activity of the treated was compared with the untreated, and the values plotted are the mean of three experiments ± SD (* represents that data is statistically significant with *p* ≤ 0.05).

Luteolin expressively decreased the methylation of crucial tumour suppressor genes in genome of HeLa cells, including *APC, BRCA1, CDH13, CDKN2, MGMT, MLH1, RARB, RASSF1,* and *TIMP3*. The methylation percentage decreased significantly after treatment with 10 and 20 µM for 48 h. The values at 10 and 20 µM luteolin treatment for 48 h were respectively: *APC* (21%, 5%); *BRCA1* (24%, 17%); *CDH1* (36%, 22%); *CDH13* (36%, 4%); *CDKN2A* (23%, 6%); *DAPK1* (23%, 13%); *FHIT* (8%, 2%); *GSTP1* (22%, 3%); *MGMT* (29%, 5%) *MLH1* (23%, 4%); *PTEN* (19%, 4%); *RARβ* (30%, 5%); *RASSF1* (28%, 10%); *SOCS1* (82%, 63%); *TIMP3* (18%, 5%);*VHL* (26%, 5%); *WIFI* (63%, 28%); and *TP73* (79.9%, 39.5%), as compared with the untreated controls, where the methylation percentage was much higher (Figure 1A).

### 2.2. Luteolin Diminishes Global DNA Methylation of HeLa Cells

Treatment of HeLa cells with 5, 10, and 20 µM of luteolin for 48 h reduced the global methylation in HeLa cells in a dose-dependent manner. Luteolin decreased global DNA methylation to 57%, 50%, and 30% at 5, 10, and 20 µM treatment for 48 h, respectively, in comparison with control (Figure 1B).

### 2.3. Luteolin Diminishes DNMT Activity in HeLa Cells

Incubation of the nuclear extract with luteolin decreased the DNMT activity in HeLa cells in a dose-dependent manner. Luteolin at 5, 10, and 20 µM reduced the DNMT activity by 35%, 57%, and 66%, respectively, in comparison with the untreated control (Figure 1C).

### 2.4. Luteolin Modulates Migration/Inflammation Related and Tumour Suppressor Genes

**Figure 2:**
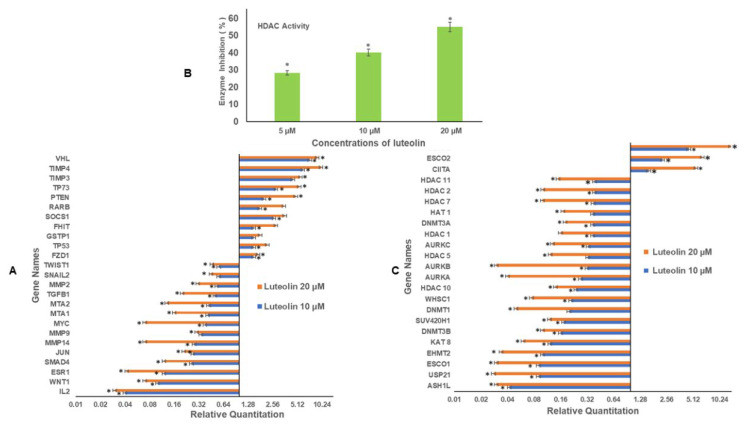
(**A**). Luteolin modulated the expression of various TSGs and migration related genes in a dose-dependent manner. The TSGs were upregulated, whereas inflammatory and migration-related genes were downregulated. (**B**). Luteolin inhibited HDAC activity in HeLa cells in a concentration-dependent manner in comparison with the untreated control. The values were taken as mean of three experiments ± SD (* represents that data is statistically significant with *p* ≤ 0.05). (**C**). Treatment of HeLa cells with luteolin at 10 and 20 µM for 48 h modulated the expression of epigenetic enzymes in a dose-dependent manner (* represents that data is statistically significant with *p* ≤ 0.05).

The decreased methylation percentage of various tumour suppressor genes after luteolin treatment was further verified by checking the expression of these genes using quantitative RT PCR. It was observed that luteolin increased the expression of various TSGs and the relative quantity (RQ) values after 10 and 20 µM treatment of HeLa cells for 48 h, respectively, for *VHL* (RQ 7, 9); *TIMP3* (RQ 4.5, 5.6); *TIMP4* (RQ 6, 10); *PTEN* (RQ 2, 4.0); *RARB* (RQ 1.78,3.5); *SOCS1* (RQ 2.65, 3.6); *FHIT* (RQ 1.5, 2.8); and *GSTP1* (RQ 1.5, 1.8) and at the same time decreased the expression of various migration related genes such as *MMP2* (RQ 0.53, 0.31); *MMP9 (*RQ 0.34, 0.38); *MMP14 (*RQ 0.28, 0.07*); SNAIL2* (RQ 0.56, 0.45)*; SMAD4* (RQ 0.26, 0.12); *MTA1 (*RQ 0.41,0.16)*; MTA2* (RQ 0.42, 0.13); and *TGFB1* (RQ 0.50, 0.20), as well as oncogenes *JUN* (RQ 0.27, 0.21) and MYC (RQ 0.38, 0.07) (Figure 2A).

### 2.5. Luteolin Decreases HDAC Activity in HeLa Cells

Histone deacetylation leads to heterochromatin and thus reduced gene expression, so the activity of histone deacetylases was analysed after direct incubation of nuclear extracts with different concentrations of luteolin. Incubation of the nuclear extract with 5, 10, and 20 µM luteolin led to decreased HDAC activity by 28%, 40%, and 55%, respectively, as compared with the controls, as shown in Figure 2B.

### 2.6. Luteolin Treatment Modulates the Expression of Chromatin-Modifying Genes

Luteolin treatment of HeLa cells modifies the expression of various chromatin-modifying enzymes. Luteolin treatment downregulated the chromatin-modifying enzymes, and the RQ values after treatment of 10 and 20 µM for 48 h, respectively, were DNMT*1* (RQ 0.20, 0.05), *DNMT3A* (RQ 0.37, 0.18), and *DNMT3B* (RQ 0.16, 0.10), where downregulated; additionally, *HDAC1* (RQ 0.37, 16)*, HDAC7* (RQ 0.38, 0.10), *HDAC2* (RQ 0.33, 0.12), *HDAC11* (RQ 0.39, 0.15), *HDAC10* (RQ 0.24, 0.14) *EHMT2* (RQ 0.10, 0.03)*, ESCO1* (0.09, 0.03), *AURKA (*RQ 0.27, 0.04), *AURKB* (RQ 0.32, 0.03), *AURKC* (RQ 0.33, 0.13), KAT*8* (RQ 0.12, 0.06), and *HAT1* (RQ 0.37, 0.17) were all downregulated after treatment of the HeLa cells with luteolin 10 and 20 µM for 48 h. However, *ESCO2* (RQ 2.30, 6.5) (acetyl transferase), *CIITA* (RQ 1.6, 5.5) (class II trans activator), and *SETD2* (methyl transferase) were upregulated. Luteolin also downregulated histone methyl transferases such as *ASH1L* (RQ 0.04, 0.03); *WHSC1 (*RQ 0.21, 0.08)*, SU2V40H1* (RQ 0.17, 0.12), and ubiquitin enzyme USP1 (Figure 2C). *CIITA* regulates the transcription of major histocompatibility complex (MHC) class I and II genes.

### 2.7. Luteolin Reduces the HAT Activity in a Dose-Dependent Manner

**Figure 3:**
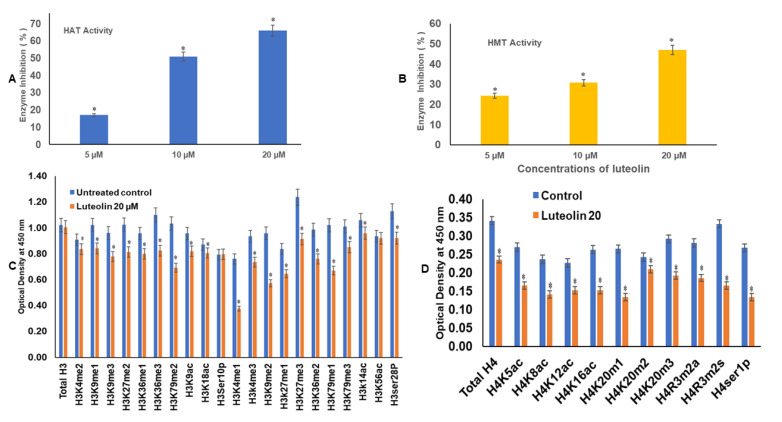
(**A**). Luteolin treatment of 5, 10, and 20 µM decreased the HAT activity in comparison with the untreated control. The plots were made as mean of three experiments ± SD ((* represents that data is statistically significant with *p* ≤ 0.05). (**B**). Luteolin treatment decreased HMT H3K9 enzyme activity in HeLa cells with respect to the untreated control. The experiment was performed three times and the values were taken as mean ± SD (* represents that data is statistically significant with *p* ≤ 0.05). (**C**). Luteolin 20 µM treatment for 48 h modulates the H3acetylation and methylation histone marks in HeLa cells, as compared with the untreated controls. (**D**). Luteolin 20 µM treatment for 48 h modulates the H4 acetylation and methylation histone marks, as compared with the untreated controls.

Luteolin at 5, 10, and 20 µM treatment decreased the HAT activity in HeLa cells, as compared with the untreated controls. On incubation of the nuclear extracts with different concentrations of luteolin, such as 5, 10, and 20 µM, there was a decrease in HAT activity by 17%, 51%, and 66%, respectively, in comparison with the untreated controls (Figure 3A).

### 2.8. Luteolin Decreases the HMT H3K9 Enzyme Activity in HeLa Cells

Histone methyl transferase HMT H3K9 enzyme causes mono, di-, and tri- methylation at histone 3 lysine 9. These marks are repressors of transcription. H3K9 methylation and DNA methylation are cooperative epigenetic changes that complement each other and are responsible for silencing of the promoters of several genes. Luteolin treatment leads to inhibition of HMT by 24%, 31%, and 47% after incubation of the nuclear extract with 5, 10, and 20 µM (Figure 3B), respectively. The inhibition percentage was obtained by comparing treated sample activity with the control sample activity.

### 2.9. Luteolin Modulated H3 and H4 Histone Marks

Luteolin decreased the expression of H3K9me1, H3K9me2, H3K9me3, H3K27me1, H3K36me2, H3K36me3, H3K79me1, and H3K79me3 marks after treatment of HeLa cells with 20 µM for 48 h; similarly, the H3 acetylation marks were diminished after treatment with the 20 µM of luteolin (Figure 3C). H3K9ac and H3K18ac were reduced likewise, and the acetylation marks at H4 were also modulated, including H4K5ac, H4K8ac, H4K12ac, and H4K16ac. H4 methylation marks including H4K20m1, H4K20m2, and H4K20m3 were decreased after chrysin treatment (Figure 3D). Phosphorylation marks of H3ser28p, H4ser1p, H4R3m2a, and H4Rm2s were also decreased after treatment of HeLa cells with luteolin 20 µM for 48 h (Figure 3C,D).

### 2.10. Luteolin Modulates Protein Expression of Genes Related to Migration, Inflammation, and TSGs

**Figure 4:**
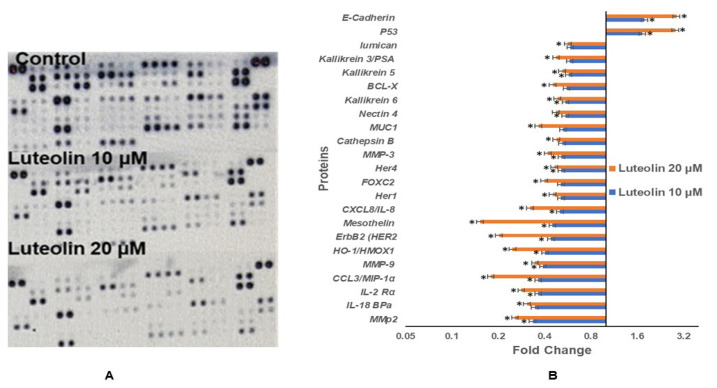
Luteolin treatment modulates the proteins related to migration and inflammation in a dose-dependent manner. (**A**). The blots show expression of different proteins. (**B**). Fold change in different proteins related to migration and inflammation (* represents that data is statistically significant with *p* ≤ 0.05).

Proteome profiler-based quantitation of proteins involved in proliferation, migration, and various cellular events showed modulation after luteolin treatment, consistent with the mRNA expression. Luteolin resulted in downregulation of expression of various proteins related to migration and inflammation in HeLa cells, and fold changes (FC) after treatment with 10 and 20 µM for 48 h are given, respectively, for MMP2 (FC 0.33, 0.26), MMP3 (FC 0.51, 0.42), MMP9 (FC 0.39, 0.35), and mesothelin (FC 0.45, 0.08), MUC1 (FC 0.53, 0.37); inflammatory proteins such as CCL3/MIP-1α (FC 0.36, 0.06), IL-18 BPa (FC 0.35, 0.31), CXCL8/IL-8 (FC 0.50, 0.32), and IL-2 Rα (FC 0.36, 0.28); and oncogenes such as HER1 (FC 0.51, 0.46) and HER4 (FC 0.51, 0.45). Genes related to cell proliferation, growth, and apoptosis such as BCL-X (FC 0.55, 0.45), HO-1/HMOX1 (FC 0.40, 0.25), Kallikrein6 (FC 0.55, 0.48), Kallikrein 3/PSA (FC 0.58, 0.48) were reduced. P53 (FC 1.7, 2.8) and E-cadherin (FC 1.8, 2.9) were upregulated (Figure 4A,B). Modulation of protein expression was well correlated with the transcript-level expression of inflammation and migration-related proteins.

### 2.11. Luteolin Repressed Colony Formation and Migration of HeLa Cells

**Figure 5:**
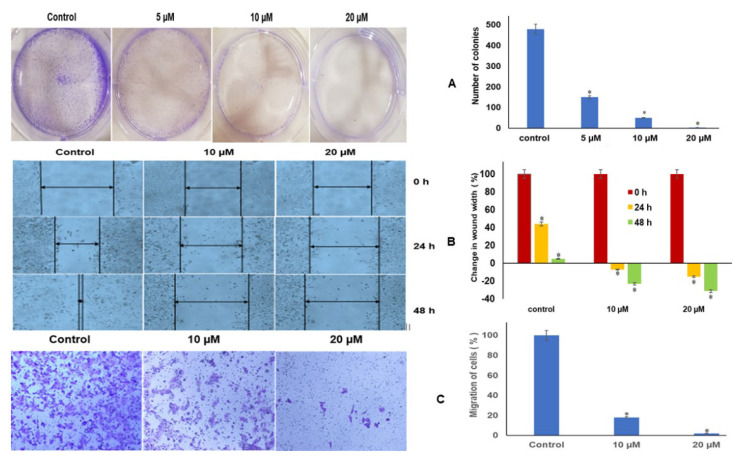
(**A**). Luteolin treatment inhibits colony formation in a dose-dependent manner with almost no colonies at 20 µM luteolin treatment for 48 h. The values plotted are mean of three experiments ± SD (* represents that data is statistically significant with *p* ≤ 0.05). (**B**). Luteolin treatment inhibits migration of HeLa cells in a dose-dependent manner, as compared with control wells, wherein almost complete wound closure could be seen after 48 h. (**C**). The luteolin-treated HeLa cells depicted significant decrease in cell migration using transwell inserts. Microscopic images at 10× magnification showing migrated cells in control and the migration at 10 and 20 µM of luteolin treatment for 48 h.

Luteolin inhibits colony formation in HeLa cells. In untreated control, a plating efficiency of 94% was observed, and at 5 µM and 10 µM luteolin treatment for 48 h, 150 and 50 colonies were seen, and at 20 µM, almost no colonies were formed (Figure 5A). However, the number of cells in untreated colonies was almost 10 to 20 cells per colony, whereas in treated colonies, the size was only 5–6 cells per colony, indicating that luteolin significantly hindered colony formation and induced the cytostatic behaviour in HeLa cells.

### 2.12. Luteolin Inhibits Migration Capacity of HeLa Cells

The migration inhibitory effect of luteolin was further confirmed by transwell assay. The migration of control cells was used to compare the migration in treated samples. The width of the wound (in cm) gradually increased in the wells treated with 10 µM for 24 h and 48 h of luteolin by 7% and 23%, respectively, and increased by 15% and 31% after 20 µM treatment for 24 h and 48 h, respectively, while the control wells exhibited the inverse, with width decreasing in a time-dependent manner, by an average of 45% by 24 h and complete closure at 48 h (Figure 5B). The migration inhibitory effect of luteolin was further confirmed by transwell assay. The migration of control cells was used to compare the migration in treated samples. Luteolin decreased the migration capacity of HeLa cells to 18% and 2% at 10 and 20 µM treatment for 48 h in comparison with the control (Figure 5C).

## 3. Discussion

Cancer is one of the main causes of mortality globally, and metastasis plays a major role in prognosis of the disease. The key enzymes involved in metastasis are the metalloproteases, and these can be potential targets for inhibition of metastasis [29]. Cancer, being a complex disease, is considered to be a manifestation of both genetic and epigenetic modulations, resulting in altered expression of genes related to proliferation, apoptosis, cell cycle, and migration [30,31] Epigenetic modulations in cancer cells include global DNA hypomethylation and hypermethylation of specific promoter regions of tumour suppressor genes (TSGs) and genes related to apoptosis and cell migration [30,32]. In spite of the fact that synthetic inhibitors showed promising results in alteration of these epigenetic modifications—owing to poor oral availability, toxicity, and lack of specificity—the ongoing search for compounds that are more specific and less toxic continues [32,33]. Interestingly, polyphenols including flavonoids have shown a number of biological activities such as antiviral, antioxidant, and pro-apoptotic effects both in vitro and in vivo [34,35]. Additionally, several flavonoids such as apigenin and luteolin have shown inhibitory effects on different epigenetic enzymes [32,36] and genes related to migration [20,37,38]. However not much is known about the effect of luteolin on cervical cell migration via modulation of the epigenetic pathway. Hence, this study was planned to analyse the modulation of epigenome after luteolin treatment and to study the effect of luteolin on migration, inflammation, and growth of HeLa cells.

Luteolin has displayed anticancer effects such as induction of apoptosis and induction of cell cycle in different cell lines [39,40]. It has shown induction of apoptosis via inhibition of E6/E7 viral proteins in cervical cancer cells [40]. Similar reports of induction of apoptosis and inhibition of various pathways in HeLa cells after luteolin treatment were visualised in our earlier study [24], wherein it was seen that luteolin caused differential cytotoxicity towards HeLa cells in comparison with the lymphocytes in the concentration range (1–40 µM). There were no significant effects of the used luteolin concentration on lymphocytes [24]. The cell viability percentages at 10 and 20 µM were 70% and 50%, respectively. These two sublethal doses (10 and 20 µM) were taken for further studies. Luteolin inducted cell cycle arrest and induced apoptosis by decreasing the expression of AKTs, cyclins, CDK2, Bcl-2, and MCL1, as well as increasing the expression of Bax, caspase-3, 8, and 9, and p21 [24]. However, different levels of cytotoxicity have been observed in different cell lines after luteolin treatment [25,26,41,42]. Luteolin has shown selective cytotoxicity towards hepatocytes of hepatocellular carcinoma (HCC) rats, as compared with normal hepatocytes [43]. Additionally, some reports have attributed anticancer effect of luteolin to being sensitive against drug resistant cancer cells [44].

Interestingly some reports have shown inhibition of metastasis in colorectal cancer (HT-29 and SW480 and downregulation of MMP 2, MMP 3, and MMP 9 via inhibition of the AKT/PI3K pathway in melanoma cells (A375 cells) after luteolin treatment [37,38]). Luteolin has depicted increased expression of MiR-26a, which is a regulator of EZH2, and at the same time, it has inhibited EZH2 and H3K27me3, leading to self-programmed death and cycle arrest and apoptosis in prostate cancer cells [45]. To understand the epigenetic modulation by luteolin, DNA methylation and histone modulation was assessed after luteolin treatment. DNA methylation of TSGs is associated with silencing of the genes including GSTP1, BRCA1, RARB, MGMT, RASSF1A, and CDKN2A/p16 [46,47,48,49,50]. In the present study, it was found that luteolin decreased the DNA methylation percentage in TSGs (Figure 1A). These TSGs are found to be hypermethylated in cervical cancer, hence leading to loss of their functions [51]. The decrease in methylation at promoters of TSGs was further affirmed by reduction in DNMT activity by 35%, 57%, and 66% after 5, 10, and 20 µM luteolin treatment (Figure 1C). Luteolin also showed a decrease in global DNA methylation to 57%, 50%, and 30% after 5, 10, and 20 µM treatment for 48 h (Figure 1B). The decrease in methylation of TSGs led to the reactivation of the genes including TP53, GSTP1, FHIT, SOCS1, RARB, PTEN, TP73, TIMP3, TIMP4, and VHL (Figure 2A) at the transcript level and E-cadherin and Tp53 at the protein level. Similar results of decrease in DNMT activity, global DNA methylation, and reactivation of various TSGs has been demonstrated by other phytochemicals, including luteolin [26,52].

Gene expression is also modulated by histone modifications that lead to hetero chromatin or euchromatin formation. Histone modifications are mediated by epigenetic enzymes such as histone acetylases, deacetylases, methylases, and demethylases which are found to be aberrantly expressed in cancer cells [53]. Luteolin showed direct inhibition of the biochemical activity of HDAC, with a sharp decline of 28%, 40%, and 55% at concentrations of 5, 10, and 20 µM (Figure 2B). This was well corelated with the downregulation of *HDAC1, HDAC5, HDAC7, HDAC2, HDAC10,* and *HDAC11* at transcript level in a dose-dependent manner (Figure 2C). The results by other scientists on human epithelioid cancer cells have demonstrated luteolin to be a potent HDAC inhibitor [25,30]. Further, it was seen that luteolin treatment decreased the expression of HAT 1 at the transcript level (Figure 2C) and that HAT enzymatic activity also decreased progressively after luteolin treatment (Figure 3A). Biochemical activity of HMT (H3K9) (Figure 3B) likewise decreased in a dose-dependent manner up to 47% (20 µM treatment). Biochemical activity of various histone modifying enzymes was authenticated by expression analysis of the histone methyl transferases and histone acetyl transferases after luteolin treatment for 48 h (10 and 20 µM), wherein downregulation of the expression of histone methyl transferases, such as *ASH1L* (H3K36me3), *EHMT2* (H3K9me3), SUV420H1 (H3K20me2, me3), WHSC1 (H3K36me2), and acetyl transferase *ESCO1* (establishment of sister chromatid cohesion N-acetyltransferase1), KAT8 (lysine acetyl transferase 8), (phosphorylases) *AURKA, AURKB,* and *AURKC,* and upregulation of the expression of acetyl transferase *ESCO2*, *CIITA,* and *SETD2* (histone methyl transferase) was observed (Figure 2C). These enzymes have been implicated in specific stages of cell migration, cell propagation, or metastasis, as described as follows. *ESCO2* has the ability to knock down MMP2, thus inhibiting migration of cancer cells [54]. *SETD2* is a tumour suppressor leading to trimethylation of H3K36me3, which is a repressive mark [55]. Major histocompatibility complex class II is linked to better prognosis of cancer patients, and its expression on cancer cells depends on the trans activator *CIITA*, which is inactivated in colorectal and gastric cancers [56], hence attributing tumour suppressor function to CIITA [53]. *WHSC1* activates *TWIST* and thus EMT; hence, its downregulation inhibits migration [57]. Inhibition of Aurora kinases has been linked to suppression of cell propagation and metastasis [58,59] and to inhibition of the development and progress of many cancers [60,61]. ESCO1 expression is also linked to cancer progression and metastasis [62]. Hence, downregulation of *ESCO1, WHSC1, AURKA, AURKB,* and *AURKC* by luteolin points towards its antiproliferative and antimigratory role.

The decrease in HAT expression at the transcript and enzyme activity level was well corelated with of inhibition of H3 acetylation marks, including H3K9ac, H3K18ac, H3K14ac, and H3K56ac (Figure 3C). Results on the same lines have been reported with curcumin and anacardic acid, which have shown an inhibitory effect on P300 and CBP [63]. In vitro and in vivo, luteolin has demonstrated similar results of inhibition of histone acetyl transferase and H3K9 and H3K14 acetylation [64]. The H3 and H4 methylation marks are linked to transcription activation (e.g., H3 lysine 4 di, tri methylation, H3, lysine 36 trimethylation, H3lysine 79 me1/me2/me3, H4 Arginine me1, H4 lysine 20 me1) or to transcription repression (e.g., H3 lysine 27 trimethylation and H3 lysine 9 (di and tri methylation)) [65]. Luteolin treatment decreased the methylation marks such as histone 3 lysine 9 mono, di, tri methylation, histone 3 lysine 27 mono, and tri methylation, histone 3 lysine 36 mono, di, tri methylation and histone 3 lysine 36 mono, di, tri methylation as well as H3K4me1, H3K4me2, and H3K4me3. The acetylation and methylation marks at H4 including the H4K5ac, H4k8ac, H4k12ac, H4K16ac H4K20me1, H4K20me1, H4K20me2, and H4 K20me3 marks were also decreased after treatment with luteolin. Phosphorylation marks of H3ser28p and H4ser1p were downregulated too (Figure 3D). These findings further verify the decreased expression of HMTs such as *ASH1L* (H3K36me3), *EHMT2* (H3K9me3), SUV420H1 (H3K20me2, me3), WHSC1 (H3K36me2), and Aurora kinases at the transcript level (Figure 2C). There is now clear evidence that Aurora B is one of the major mitotic kinases that phosphorylates H3 at ser10 and ser28 [66]. Thus, decrease in Aurora kinase A and B explains the decrease in these H3 ser phosphorylases marks after luteolin treatment. Overexpression of Aurora kinase A is also linked to overexpression of MMP 7 and MMP 9, inhibition of Aurora kinases inhibits proliferation, migration and metastasis [58,59,67].

Interestingly, luteolin further verified an antimigration and anti-inflammatory effect by downregulation of various genes related to migration and inflammation, such as *IL2, ESR1, MMP14, MMP9, MTA1, MTA2, MMP2, SNAIL2, SMAD4,* and *TWIST1* and increased expression of PTEN, *TIMP3, TIMP4, TP73, VHL,* and *SOCS1* after luteolin treatment at 10 and 20 µM for 48 h (Figure 2A). *TIMP3, TIMP4,* and *SOCS1* are negative regulators of MMPs that regulate the migration machinery in HeLa cells. The above findings were reinforced by similar results at the protein level, wherein luteolin treatment decreased the inflammatory and migratory proteins such as MMp-2, MMP-3, HO-1/HMOX1, Her1, HER2, Her4, mesothelin, cathepsin B, MUC1, nectin 4, FOXC2, IL-18 BPa, CCL3/MIP-1α, CXCL8/IL-8, IL-2 Rα, kallikrein 6, BCL-X, kallikrein 5, kallikrein 3/PSA, and lumican and upregulated E-cadherin and P53 (Figure 4A,B). E-cadherin expression inhibits migration and is hypermethylated in cancer cells [68], and P53 is an important TSGs and has a role in the cellular processes of proliferation and migration [69]. Similar results of inhibition of migration and invasion by luteolin on melanoma and breast cancer cells were obtained by other studies [38,70]. Comparable results have been reported with apigenin, wherein it increased the expression of cadherin and decreased expression of vimentin in HCT116 and inhibited migration in MDA-MB-231 by downregulation of *SNAIL* and *N-cadherin* [71,72].

The modulation of the proliferative and migratory pathway was further verified by colony assay, scratch-wound, and invasion assays after 5, 10, and 20 µM treatment of luteolin. The number of colonies formed decreased from 480 in control to 150 and 50 at 5 and 10 µM for 48 h; however, no colonies could be seen at 20 µM treatment (Figure 5A), indicating complete cytostatic induction. In scratch wound assay, the increase in wound width was visualized by 7% and 23% at 10 µM treatment for 24 and 48 h and 15% and 31% increase in wound width at 20 µM luteolin treatment for 24 and 48 h treatment, respectively (Figure 5B). This was further supported by transwell assay, wherein luteolin decreased the migration capacity of HeLa cells to 18% and 2% at 10 and 20 µM treatment for 48 h (Figure 5C). These findings further supported the downregulation of pro-migratory (MMPs) genes and upregulation of antimigratory (*TIMPs* and *PTEN*) and antiproliferative genes. Similar results of inhibition of migration and invasion by luteolin in colorectal cells and squamous cell carcinoma cells were obtained by some authors [37,38,73].

Our study systematically demonstrates the mode of action of luteolin, which involves downregulating the expression and activity of epigenetic modulators such as DNMTs, HDACs, HMTs, and HAT, leading to consequential demethylation of promoters of TSGs and reactivation of TSGs. In addition, luteolin downregulated the expression of genes related to proliferation and migration, such as MMPS, SNAIL, MTA1, and MTA2, and upregulated inhibitors of migration such as TIMPs and TSGs such as PTEN.

## 4. Material and Methods

### 4.1. Maintenance of Cervical Cancer Calls (HeLa) and Drug Dilution

Human cervical cancer cell line (HeLa) was maintained in DMEM supplemented with 10% FBS and incubated at 37 °C. Luteolin was purchased from Sigma-Aldrich (Merck, KgaA, Darmstadt, Germany) and a stock of 69.87 mM was prepared with DMSO. Further concentration of 1mM was prepared and concentrations (1–40 µM) were made.

### 4.2. Methylation-Specific PCR (MSRE-PCR)

An EpiTect Methyl II PCR kit (catalogue number 335452, Qiagen, Germantown, MD, USA) was procured. This kit helps in the quantitation of methylated and unmethylated DNA. Restriction digestion was conducted on 1 µg of genomic DNA (treated and DMSO-treated), and the cleaved DNA in each reaction was measured using quantitative PCR. The amount of unmethylated and methylated DNA was assessed using the ^∆∆^CT technique, which compared the quantity of each reaction to that of a mock (no enzymes added) response. The gene panel (which included pre-designed primers) included tumour suppressor genes. The mean of three experiments was used to assess statistical significance (*p* < 0.05).

### 4.3. Global DNA Methylation Assay

The DNA of the treated (5, 10, and 20 µM of luteolin for 48 h) and vehicle control cells was extracted using the GenElute Mammalian Genomic DNA Miniprep Kit (Catalogue number G1N70; Sigma-Aldrich, Merck, KgaA). The quality and quantity of DNA was checked by gel electrophoresis (1% agarose gel, Catalog No. A9539, Sigma-Aldrich, Merck, KgaA) and spectrophotometry using Nanodrop 2000 (Thermo Scientific™, Waltham, MA, USA), respectively. The MethylFlash™ Methylated DNA Quantification Kit (Catalogue number: P-1034; Epigentek, Farmingdale, NY, USA) was used for the detection of methylated DNA by 5-mC antibody, which can be assessed calorimetrically.

The treated and untreated DNA were investigated for global DNA methylation as per the protocol. Absorbance was read on an ELISA reader at 450 nm, 10 min after placement in developing solution. Optical density values are relative to the quantity of methylated DNA, and the levels of methylation were calculated in comparison with the control. Data were taken as the average of 3 experiments ± SD. Significant differences were established at *p* ≤ 0.05.

### 4.4. Expression Analysis of TSGs

GenElute Mammalian Genomic Total RNA Kit, Sigma, Merck, KgaA, was procured. The manufacturer’s instructions were followed while extracting RNA from luteolin-treated HeLa (10 and 20 M for 48 h) and untreated cells. The Applied Biosystems High-Capacity cDNA Reverse Transcription Kit was then used to synthesize cDNA on the RNA. The expression of various TSGs and genes encoding various pathways was analysed using a TaqMan-based custom array (4391524). Thermo Fisher’s DataAssistTM software was used to perform the PCR array on QuantStudio3 and analyse it using the ^∆∆^CT technique. ACTB (Actin Beta) expression was used for normalization.

### 4.5. Protein Expression by Proteome Profiler Array

Proteome profiler array (catalogue no. ARY026) was procured from R&D, USA. The treated (10 and 20 µM for 48 h) and untreated cells were collected and resuspended in lysis buffer 17 (1 mL per 10^7^ cells); the lysate was prepared, and protein concentration was checked using Pierce BCA Assay (Catalogue no: 23225; Thermo-Fisher Scientific, Waltham, MA, USA). The signals produced on the membranes were quantified by chemiluminescent gel doc system (Bio-Rad Laboratories, Hercules, CA, USA) and analysed using Image Lab software (version 6.1). Significance was calculated by one-way ANOVA using SPSS software and was documented at *p* ≤ 0.05.

### 4.6. Analysis of Epigenetic Enzymes Involved in Chromatin Modification

Human Chromatin Modification Enzymes Array (Catalogue number PAHS-085Z; Qiagen, MD, USA) was used to check the expression of the genes such as DNMTs, HMTs, HATS, and HDMs. RNA at a concentration of 1 µg was used to synthesize cDNA, and it was diluted to 1350 µL with nuclease free water, and an equal amount of RT2 SYBR^®^ Green qPCR Master mix (Catalogue number: 330504; Qiagen, MD, USA) was added to this. From this mixture, 25 µL was poured into each well of an array plate having pre-defined primers, and the plate was run on ABI Quant Studio 3. The normalization was performed using GAPDH as the housekeeping gene, and fold change was calculated by comparing treated samples with untreated controls. The statistical significance was calculated, and the significance was established at *p* ≤ 0.5.

### 4.7. Nuclear Extract Preparation

Nuclear extracts from HeLa cells were prepared using EpiQuik^TM^ nuclear extraction kit (Catalogue number OP-0002; Epigentek, NY, USA) as per the manufacturer’s protocol. Approximately 2 × 10^6^ untreated cells were trypsinised, collected, and centrifuged at 150× *g* for 10 min. The nuclear extract was prepared and stored at −80 °C until used. The protein concentration of the nuclear extract was checked using the BCA protocol.

### 4.8. DNMT Activity Assay

Nuclear extract from untreated HeLa cells was prepared using EpiQuik^TM^ nuclear extraction kit (Catalogue number OP-0002; Epigentek, NY, USA). The EpiQuik DNMT activity assay kit (Catalogue number P-3001; Epigentek, NY, USA) was used to check the effect of the luteolin on DNMT activity. In the sample wells AdoMet (methyl group donor), nuclear extracts, and different concentrations of luteolin (5, 10, and 20 µM in assay buffer) were used to check for DNMT activity by following the protocol, whereas assay buffer was used in controls instead of the inhibitor. After incubating the plate for 2 h and developing the signal, the plate was read at 450 nm. The percentage of inhibition in comparison with control was calculated and plotted as a graph. The experiment was repeated thrice and the mean ± SD was used to plot a graph. One-way ANOVA was used to check for statistical significance, and *p* was established at ≤0.05.

### 4.9. HDAC Activity Assay

The EpiQuik HDAC activity assay kit (Catalogue number P-4002; Epigentek, NY, USA) was used to determine the effect of luteolin treatment on HDAC activity. Luteolin (5 µM, 10, and 20 µM), nuclear extract, and HDAC assay buffer were added to the assay plates and incubated for one hour at 37 °C to allow the action of the enzyme, followed by incubation with capture and detection antibody. In the controls, luteolin was substituted by assay buffer. The plate was read on an ELISA reader at 450 nm. Inhibition of HDAC activity by luteolin was calculated in comparison with control wells. Experiments were repeated thrice, and the mean ± SD was used to plot as a graph (*p* ≤ 0.05).

### 4.10. HMT H3K9 Activity Assay

The EpiQuik HMT H3K9 activity assay kit (Epigentek, NY, USA) was used to check the effect of luteolin on HMT H3K9 activity by following the manufacturer’s protocol. Briefly, to histone 3 lysine substrate-coated assay plate biotinylated substrate, assay buffer and AdoMet were added and incubated for 1.5 h at 37 °C. In the case of test samples, luteolin at 5, 10, and 20 µM was added to the wells. After developing the colour, the absorbance was read on an ELISA reader at 450 nm. The inhibition of HMT by luteolin in comparison with the controls was calculated and plotted as a graph. The results were taken as the mean of 3 experiments ± SD (*p* ≤ 0.05).

### 4.11. HAT Activity

HAT activity/inhibition kit (catalogue number P-4003, Epigentek, NY, USA) was procured. Briefly, the nuclear extracts were incubated with the substrate for HAT for 60 min, followed by washing. Luteolin concentrations were added in the test samples. The experiment was carried out as per the manufacturer’s protocol. The plate was read at 450 nm on an ELISA plate reader, and the HAT activity inhibition was calculated from the average of three experiments. The results were calculated as the mean of three experiments ± SD, (*p* ≤ 0.05).

### 4.12. Scratch-Wound Assay

To understand the antimigratory effect of luteolin, the scratch-wound assay was carried out. The protocol was adapted from our lab [74]. Briefly, almost 5 × 10^5^ cells were plated in a six-well plate and incubated at 37 °C overnight. Next day a “wound” or a cell-free line was created on a confluent monolayer of cells by scratching the monolayer with a pipette tip, and the cells were incubated in the presence of different luteolin concentrations (0, 10, and 20 µM), and the “healing” of the wound, which occurs through cell migration and growth towards the cell free zone, was monitored after 24 h and 48 h. An inverted microscope was used to capture images of the wounds in each well prior to treatment and after 24 h and 48 h of the treatment. The wound width was measured using the paint software. The change in wound width in comparison with the control was calculated. The experiment was triplicated, and results expressed ± SD (*p* < 0.05).

### 4.13. Colony Forming Assay

Approximately 2.5 × 10^5^ cells were plated in a six well plate and followed by treatment with different concentrations of luteolin (5, 10, and 20 µM) for 48 h. Treated cells were collected after 48 h treatment, counted, and plated at approximately 500 cells/well. The cells were allowed to grow for two weeks. Then, cells were fixed in 100% methanol and stained with 0.1% crystal violet. The stained colonies were counted and images were taken using an Olympus inverted microscope (Labomed, Los Angeles, CA, USA), and ImageJ software was used to count the colonies [75].

### 4.14. Trans Well Chamber Assay

Briefly, 5.0 × 10^3^ cells/well (both treated and untreated) were seeded on the upper side of the insert (8 μm pore size, BD Biosciences, Franklin Lakes, NJ, USA), and on the lower side of the insert in the 24-well plate, complete media was kept. Cells on the inner side of the insert were stained with 0.1% crystal violet, and cells on the outer side of the insert were scraped. Invasive potential of treated and untreated cells was compared, and the migrated cells were counted by using an inverted microscope (Olympus Corporation, Tokyo, Japan, 200× magnification). Graphs were plotted after repetition of the experiment three times, and the results are expressed as mean ± SD (*p* < 0.05).

### 4.15. Statistical Analysis

The results of the experiments are expressed as average ± standard deviation of three experiments. SPSS software version 21.0 was used to check the statistical significance with one-way ANOVA followed by Tukey’s HSD post hoc analysis.

## 5. Conclusions

Luteolin acts as a potent antiproliferative, antimigratory agent. It decreased the 5′CpG promoter methylation of various TSGs, leading to re-expression of these TSGs and inactivated genes related to migration and inflammation. Considering its antimigratory and epigenetics modulatory behaviours warrants its use as a potential chemopreventive agent for therapeutic use, but only after clinical trials.

## Data Availability

The datasets used and/or analysed during the present study are available from the corresponding author on reasonable request.
